# Supplementary material for: An ErbB2/c-Src axis links bioenergetics with PRC2 translation to drive epigenetic reprogramming and mammary tumorigenesis
Source: Nat Commun. 2019 Jul 1;10:2901. doi: 10.1038/s41467-019-10681-4 (PMC6603039; doi:10.1038/s41467-019-10681-4)

An ErbB2/c-Src axis drives mammary tumourigenesis through metabolically directed translational regulation of Polycomb Repressor Complex 2

Smith, H.W. et al.

Supplementary Information

Supplementary Information contains 11 Supplementary Figures

Supplementary Figure 1

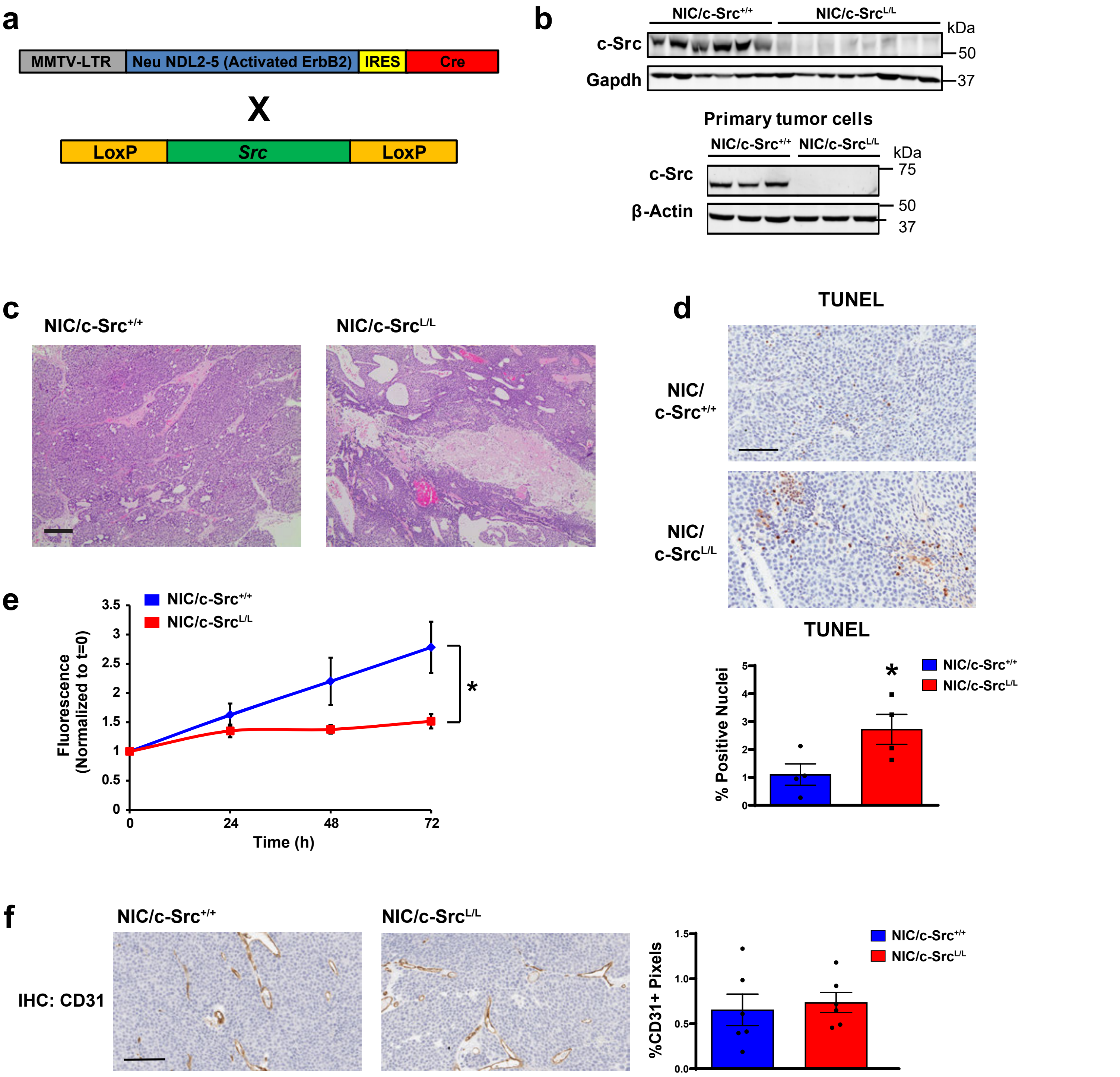

Supplementary Figure 1. c-Src ablation increases tumour cell death and impairs tumour cell proliferation (a) Schematic showing the MMTV-NIC transgene, which was crossed into the conditional c-Src knockout background. **(b)** Immunoblot showing c-Src levels in NIC/c-Src$^{+/+}$ and NIC/c-Src$^{L/L}$ tumors and primary cell cultures with depletion of contaminating stroma. **(c)** H&E staining of NIC/c-Src$^{+/+}$ and NIC/c-Src$^{L/L}$ tumors. Images are representative of eight independent tumors of each genotype. Scale bar represents 200 μm. **(d)** Terminal deoxynucleotidyl transferase dUTP nick end labeling (TUNEL) was performed on tumor samples from 5 independent mice of each genotype. TUNEL positive nuclei were quantified using an Aperio XT slide scanner and associated software and expressed as the percentage of total nuclei counted (mean +/- SEM, minimum 10 000 total nuclei analyzed, *p < 0.05). Scale bar represents 100 μm. **(e)** Proliferation of control and c-Src-deficient tumor cells was analyzed *in vitro*. *p < 0.05; two-tailed Student's t-test at 96h. **(f)** Control and c-Src-deficient tumours (n=5 per genotype) were stained for CD31 expression, as a marker of endothelial cells, by immunohistochemistry (IHC). Bar chart shows quantification of CD31 positive pixels (mean +/- SEM, no significant difference; unpaired, two-tailed Student's t-test).

Supplementary Figure 2

a

Pathways Up-regulated in NIC/c-Src$^{L/L}$ Tumours

KEGG Term	P-value
ECM-receptor interaction	6.00824E-06
PI3K-Akt signaling pathway	0.000288199
Protein digestion and absorption	0.000372628
Focal adhesion	0.000423989
Small cell lung cancer	0.001118713
Mucin type O-Glycan biosynthesis	0.00215834
Amoebiasis	0.003197731
TNF signaling pathway	0.006001644
VEGF signaling pathway	0.009919752
Metabolic pathways	0.01449648
**REACTOME Term**	**P-value**
Collagen biosynthesis and modifying enzymes	0.000106087
Assembly of collagen fibrils and other multimeric structures	0.000197356
Collagen formation	0.000233067
Extracellular matrix organization	0.000388798
O-linked glycosylation of mucins	0.00079463
Pyruvate metabolism and Citric Acid (TCA) cycle	0.000544914
Metabolism of lipids and lipoproteins	0.00169113
Regulation of pyruvate dehydrogenase (PDH) complex	0.000639444
Pyruvate metabolism	0.001132249
Sphingolipid de novo biosynthesis	0.002869525

GO Biological Process	P-value
cell-matrix adhesion	1.78922E-05
protein localization to membrane	2.96936E-05
extracellular matrix organization	7.28996E-05
negative chemotaxis	0.000125132
cortical cytoskeleton organization	0.000232467
**GO Cellular Component**	**P-value**
focal adhesion	3.48575E-09
specific granule membrane	0.000445184
Golgi membrane	0.000988779
cytoskeleton	0.001183202
specific granule	0.001197351
**GO Molecular Function**	**P-value**
cadherin binding	8.5959E-05
integrin binding	0.00013272
type II transforming growth factor beta receptor binding	0.001357047
ubiquitin-like protein ligase binding	0.001814577
type I transforming growth factor beta receptor binding	0.001990184

b

Pathways Down-regulated in NIC/c-Src$^{L/L}$ Tumours

KEGG Term	P-value
Glycosaminoglycan biosynthesis - keratan sulfate	0.019255109
Synaptic vesicle cycle	0.036713509
**REACTOME Term**	**P-value**
Retrograde neurotrophin signalling	0.012829062
Catabolism of glucuronate to xylulose-5-phosphate	0.014216836
Recycling pathway of L1	0.017697906
EGFR downregulation	0.020146695
CDO in myogenesis	0.025642744
Myogenesis	0.025642744
Serotonin Neurotransmitter Release Cycle	0.031658681
peptide receptors bind formyl peptides and many other	0.03681012
Sialic acid metabolism	0.039101155
Formation of annular gap junctions	0.046116818

GO Biological Process	P-value
regulation of interleukin-2 production	0.009797623
positive regulation of T cell cytokine production	0.012829062
clathrin coat assembly	0.019255109
regulation of protein K63-linked ubiquitination	0.028334819
response to gonadotropin	0.028334819
**GO Cellular Component**	**P-value**
clathrin vesicle coat	0.028334819
clathrin coat	0.028334819
IPAF inflammasome complex	0.028334819
AIM2 inflammasome complex	0.03681012
**GO Molecular Function**	**P-value**
U4 snRNA binding (GO:0030621)	0.001872554
cytochrome-b5 reductase activity, acting on NAD(P)H	0.028334819
5'-3' exodeoxyribonuclease activity	0.028334819
azole transmembrane transporter activity	0.028334819
oxidoreductase activity, acting on the CH-NH group of donors, NAD or NADP as acceptor	0.036526301

c

Transcriptional Regulators of Genes Up-regulated in NIC/c-Src$^{L/L}$ Tumours and Cell Lines

Factor	Mammary Tumours	Breast Cancer Cell Lines
CEBPB	ChEA, ENCODE	IPA, ChEA, ENCODE
EZH2	ChEA, ENCODE	IPA, ChEA, ENCODE
TP53	IPA, ChEA	IPA, ChEA, ENCODE
STAT3	IPA, ChEA	IPA, ChEA
SMARCA4	IPA, ChEA	IPA
CTNNB1	IPA	IPA, ChEA
EBF1	ChEA	IPA, ChEA
NFE2L2	ChEA	IPA, ChEA
PPARG	ChEA	IPA, ChEA
SUZ12	ChEA, ENCODE	ChEA

d

Pathway Analysis: Genes with H3K27me3 +/- 20kb of the TSS in NIC/c-Src$^{+/+}$ Tumours

KEGG Term	P-value
Butirosin and neomycin biosynthesis	0.016634753
Phenylalanine, tyrosine and tryptophan biosynthesis	0.016634753
Galactose metabolism	0.037178818
Oxytocin signaling pathway	0.0379684
**REACTOME Term**	**P-value**
Phase 2 - plateau phase	0.000649852
NCAM1 interactions	0.004407629
Phase 1 - inactivation of fast Na+ channels	0.005125029
Na+/Cl- dependent neurotransmitter transporters	0.007596186
Phase 0 - rapid depolarisation	0.010411874
LGI-ADAM interactions	0.019724272
Cardiac conduction	0.029551743
SLC transporter disorders	0.033005777
Neurofascin interactions	0.033005777
Amine compound SLC transporters	0.037178818

GO Biological Process	P-value
neurotransmitter receptor transport	0.000848695
action potential	0.002164555
neurotransmitter receptor transport, postsynaptic endosome to lysosome	0.003672752
forebrain neuron differentiation	0.003672752
postsynaptic neurotransmitter receptor diffusion trapping	0.005335622
**GO Cellular Component**	**P-value**
juxtaparanode region of axon	0.000558875
main axon	0.013832878
cation channel complex	0.021879673
ionotropic glutamate receptor complex	0.026573056
L-type voltage-gated calcium channel complex	0.042783451
**GO Molecular Function**	**P-value**
voltage-gated cation channel activity	2.37628E-05
voltage-gated calcium channel activity	0.000986368
sodium:amino acid symporter activity	0.001717503
calcium channel activity	0.002160893
anion:cation symporter activity	0.00240075

e

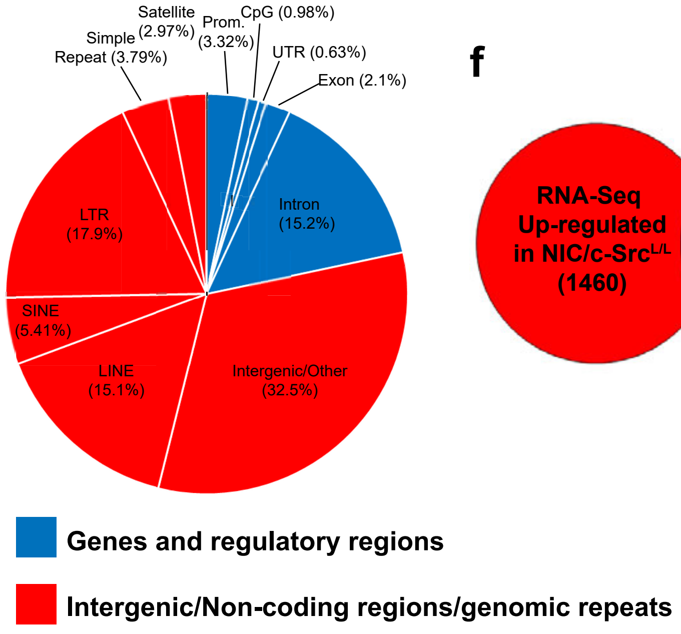

f

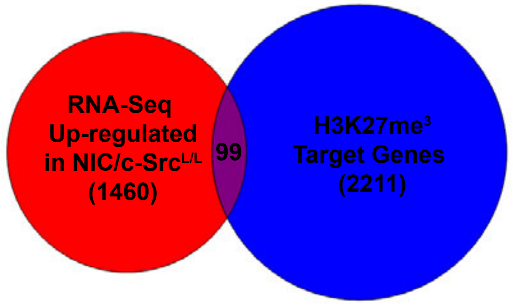

g

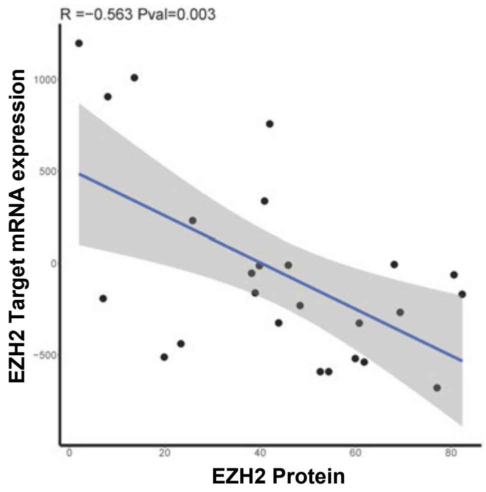

Supplementary Figure 2. Transcriptomic and epigenomic alterations in c-Src-deficient ErbB2+ mammary tumours. (a-b) Genes up-regulated (a) and down-regulated (b) in NIC/c-Src$^{L/L}$ vs. NIC/c-Src$^{+/+}$ tumors were analyzed for enrichment in specific molecular pathways using the Kyoto Encyclopedia of Genes and Genomes (KEGG), the REACTOME database and the Gene Ontology (GO) database. For KEGG and REACTOME, the top ten significantly enriched pathways ($p < 0.05$), ranked by p-value, are shown. Less than 10 entries in the table indicates that fewer than ten pathways were identified as statistically significant. For GO, the top five significantly enriched terms ($p < 0.05$), ranked by p-value, are shown. **(c)** Differentially expressed genes (up-regulated and down-regulated) in NIC/c-Src$^{L/L}$ vs, NIC/c-Src$^{+/+}$ tumors (microarray) and cell lines (RNA-Seq) were analyzed for signatures of transcriptional regulators by cross-referencing with the ChEA and ENCODE databases and by using Ingenuity Pathway Analysis (IPA). The table shows potential transcriptional regulators of these differentially expressed genes that were identified in both sample types, where at least two independent methods identified the same regulator in at least one sample type (tumours and/or cell lines). No candidate regulators of genes down-regulated in NIC/c-Src$^{L/L}$ tumours met these criteria, hence the table shows only candidate regulators of genes up-regulated in NIC/c-Src$^{L/L}$ tumours. Red boxes highlight components of PRC2. **(d)** Pathway analysis as in (a-b) was applied to genes where H3K27me^3 ChIP-Seq identified peaks within 20kb of the transcriptional start site (TSS) in NIC/c-Src$^{+/+}$ cells. **(e)** Pie chart indicates the distribution of H3K27me^3 peaks among various genomic features. Blue segments indicate genes and gene regulatory regions, red segments indicate non-coding regions including transposable elements and repeat regions. **(f)** Venn diagram indicates overlap between H3K27me3 peaks in NIC/c-Src$^{+/+}$ cells and genes transcriptionally up-regulated in NIC/c-Src$^{L/L}$ cell lines. **(g)** The transcript level of a 40-gene signature comprised of genes targeted by PRC2 (based on ChIP-Seq in two independent NIC/c-Src$^{+/+}$ cell lines) that were transcriptionally activated in NIC/c-Src$^{L/L}$ cells was correlated with EZH2 protein as in Figure 4g (R^2 and p values, Pearson's correlation analysis).

Supplementary Figure 3

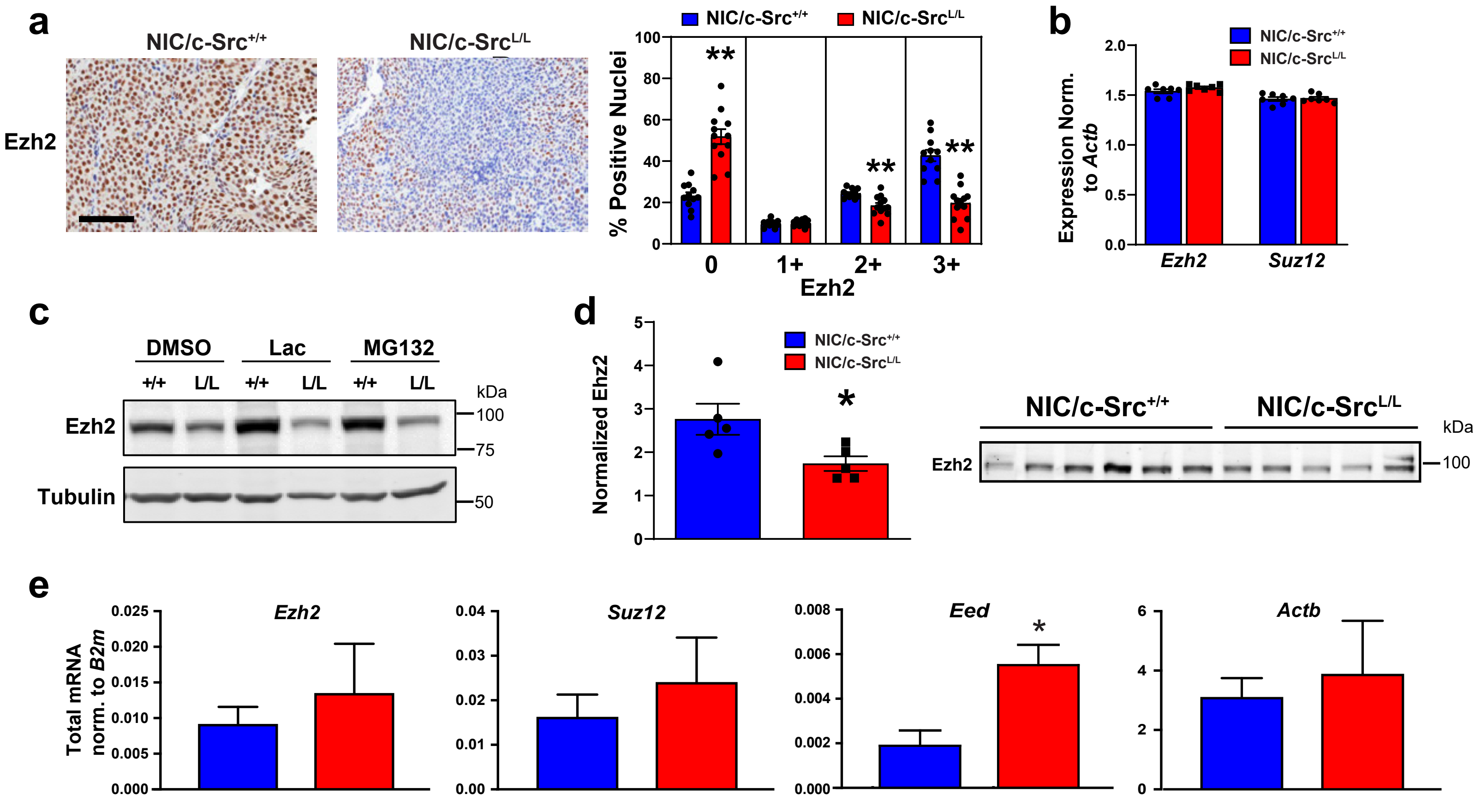

Supplementary Figure 3. c-Src ablation affects translation of PRC2 components independently of effects on mRNA expression and protein stability. (a) NIC/c-Src$^{+/+}$ and NIC/c-Src$^{L/L}$ tumors were stained for Ezh2 using immunohistochemistry. Nuclear staining intensity was scored on a scale of 0-3 using Aperio image analysis software (mean +/- SEM, minimum 10000 nuclei counted per tumor, *$p < 0.05$, **$p < 0.01$; unpaired, two-tailed Student's t-test). Images are representative of tumors from three independent mice of each genotype. Scale bar represents 50 µm. **(b)** qRT-PCR analysis of *Ezh2* and *Suz12* mRNA levels normalized to *Actb* (β-actin) in tumor samples from seven NIC/c-Src+/+ and seven NIC/c-Src$^{L/L}$ mice. Data are mean +/- SEM. An unpaired two-tailed t-test indicated no significant differences. **(c)** Immunoblot analysis of Ezh2 levels in control NIC tumor cells (+/+) and c-Src-deficient cells (L/L) treated for 6h with the proteasome inhibitors lactacystin A (Lac - 1 µM) and MG132 (10 µM). **(d)** Immunoblot analysis of Ezh2 protein levels in NIC tumor cells from Figure 2 (B-C). Bar chart shows Ezh2 fluorescent signal (Li-COR Odyssey) normalized to the total protein loaded in each lane (Figure 2B) (*$p < 0.05$, unpaired, two-tailed Student's t-test). **(e)** qRT-PCR analysis of *Ezh2*, *Suz12*, *Eed*, and *Actb* mRNA levels normalized to β2-microglobulin (*B2m*) in NIC/c-Src$^{+/+}$ and NIC/c-Src$^{L/L}$ cell lines (Mean +/- SEM, 3 biological replicates per genotype). The only statistically significant difference identified was an increase in *Eed* mRNA expression in c-Src-deficient cells (unpaired two-tailed Student's t-test).

Supplementary Figure 4

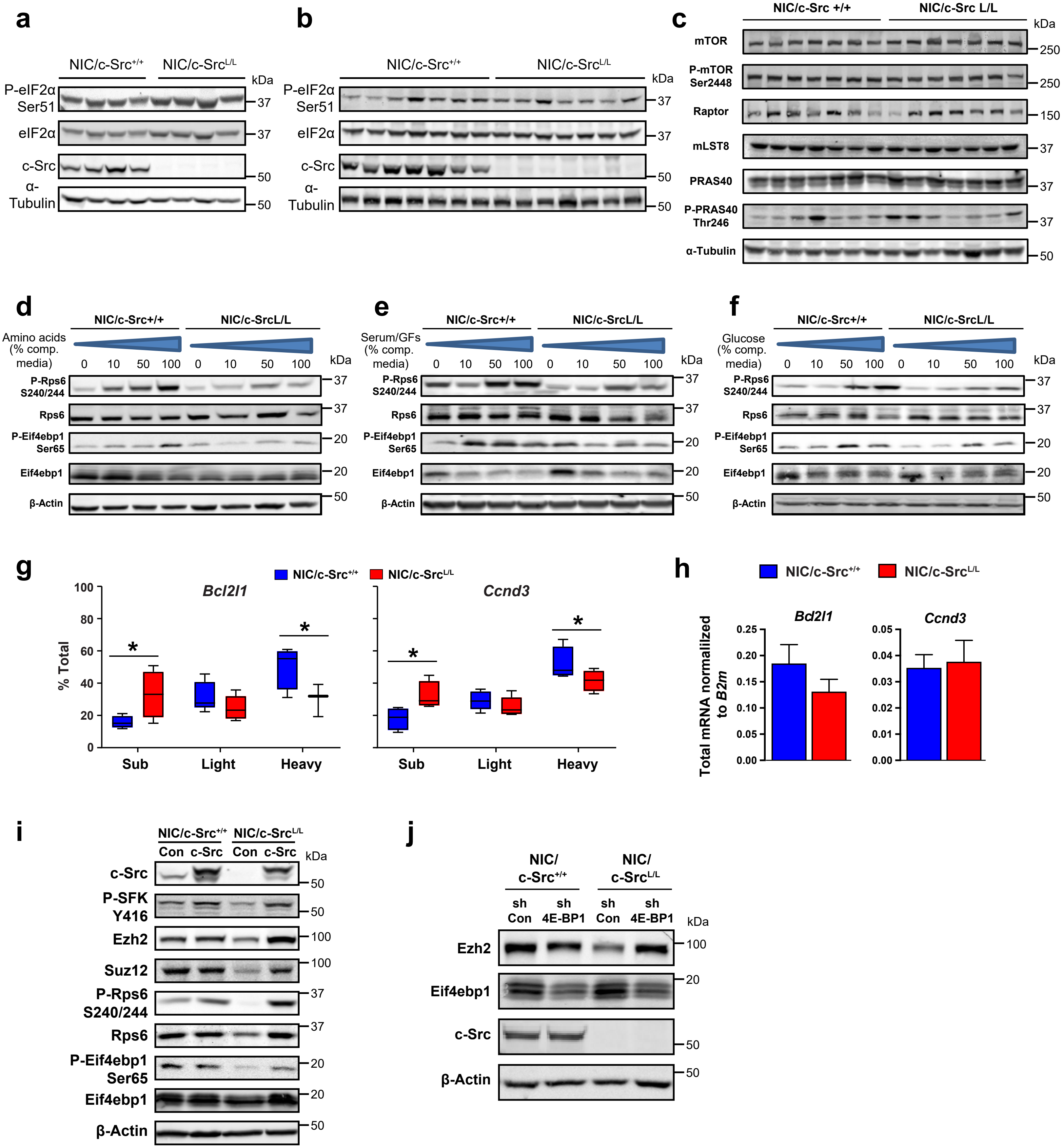

Supplementary Figure 4. Suppression of mTORC1 activity and mTORC1-dependent translation in c-Src-Deficient ErbB2+ mammary tumours. (a-b) NIC/c-Src$^{+/+}$ and NIC/c-Src$^{L/L}$ cell lysates (a) or tumour lysates (b) were immunoblotted to detect the total and phosphorylated forms of Eif2α as well as c-Src. α-Tubulin was used as a loading control. **(c)** The expression and phosphorylation (for mTOR and PRAS40) of mTORC1 components was NIC/c-Src$^{+/+}$ and NIC/c-Src$^{L/L}$ tumours by immunoblotting (n=7 per genotype). **(d-f)** NIC/c-Src$^{+/+}$ and NIC/c-Src$^{L/L}$ cell lines were starved of amino acids for 1h (d), starved of serum and growth factors overnight (e) or starved of glucose overnight (f) to inactivate mTORC1 and then stimulated for 30 min with increasing levels of these nutrients or factors, representing 10%, 50% and 100% of the levels in their complete growth media (DMEM with supplements - see Methods). Reactivation of mTORC1 was monitored by immunoblotting. Data are representative of three biological replicates per genotype and the experiment was performed twice. **(g)** qRT-PCR analysis of known mTORC1-dependent mRNAs (*Bcl2l1*, *Ccnd3*) isolated from polysome profiling of NIC/c-Src$^{+/+}$ and NIC/c-Src$^{L/L}$ tumor cell lines (mean +/- SEM, 5 biological replicates per genotype, *$p < 0.05$; unpaired, two-tailed Student's t-test). **(h)** qRT-PCR analysis of *Bcl2l1* and *Ccnd3* mRNA levels normalized to β2-microglobulin (*B2m*) in NIC/c-Src$^{+/+}$ and NIC/c-Src$^{L/L}$ cell lines (Mean +/- SEM, 5 biological replicates per genotype). No statistically significant differences were identified (unpaired two-tailed Student's t-test). **(i)** NIC/c-Src$^{+/+}$ and NIC/c-Src$^{L/L}$ cell lines were transduced with retroviruses expressing c-Src or empty vector control and immunoblotted to detect the expression levels of the indicated proteins. **(j)** Immunoblot showing Ezh2, Eif4ebp1 and c-Src levels in NIC/c-Src$^{+/+}$ and NIC/c-Src$^{L/L}$ cell lines transduced with lentiviruses bearing shRNA against *Eif4ebp1* (sh4E-BP1) or LacZ (shCon).

Supplementary Figure 5

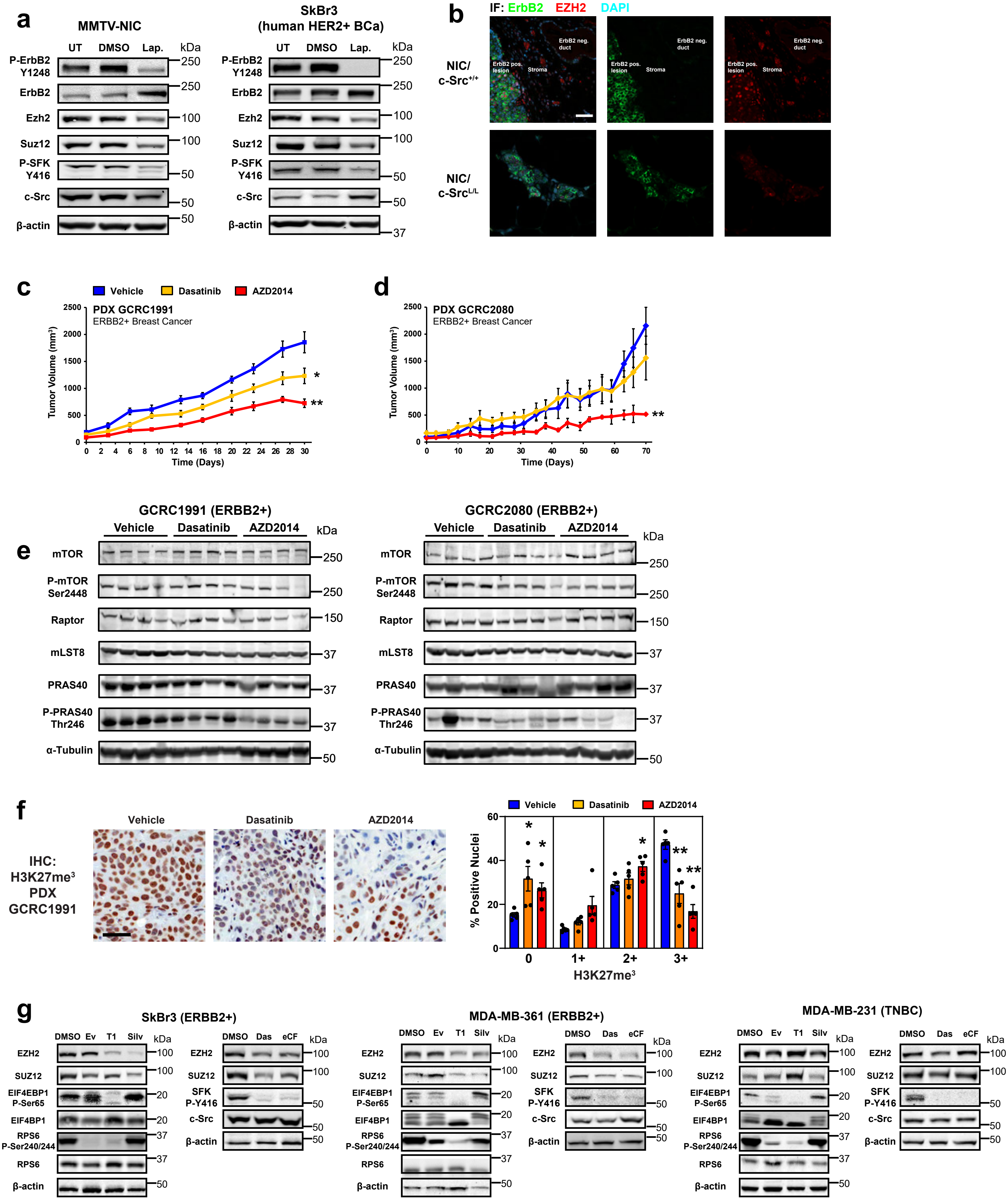

Supplementary Figure 5. Regulation of PRC2 component protein expression by ErbB2/c-Src/mTORC1 signaling in GEMMS, PDX models and human breast cancer cell lines. (a) ErbB2 and SFK activity as well as Ezh2 protein levels were determined by immunoblotting in murine (MMTV-NIC) and human (SkBr3) ErbB2+ breast cancer cell lines following 24h of treatment with the ErbB2 tyrosine kinase inhibitor lapatinib (Lap.) or vehicle control (DMSO). UT denotes untreated cells. **(b)** IF analysis of ErbB2 and Ezh2 protein levels in mammary tissue from 20 week-old NIC/c-Src$^{+/+}$ and NIC/c-Src$^{L/L}$ mice. Representative images from five mice per genotype. Scale bars represent 50 µm. **(c-d)** ERBB2+ breast cancer patient-derived xenografts GCRC1991 (c) and GCRC2080 (d) were orthotopically implanted into NOD/SCID/gamma (NSG) mice which were treated with the SFK inhibitor Dasatinib (n=7), the ATP-competitive mTOR kinase inhibitor AZD2014 (n=7) or vehicle (n=6). Tumor growth was determined by caliper measurements at the indicated times following initiation of treatment (*$p < 0.05$, **$p < 0.01$; one-way ANOVA with Tukey's post-test). **(e)** PDX tumour samples from (d) were immunoblotted to examine the expression and phosphorylation (for mTOR and PRAS40) of mTORC1 component proteins. **(f)** H3K27me^3 levels were analyzed in tumours from PDX GCRC1991 treated as in (c) using IHC (as in Figure 1e). Scale bar represents 50 µm. Bar chart shows nuclear staining intensity scored on a scale of 0-3 using Aperio image analysis software (mean +/- SEM, minimum 10000 nuclei counted per tumour, *$p < 0.05$, **$p < 0.01$; unpaired, two-tailed Student's t-test vs. vehicle control). **(g)** Human ERBB2+ (SkBr3 and MDA-MB-361) and triple-negative (MDA-MB-231) breast cancer cell lines were treated with the indicated inhibitors for 24h (Ev: everolimus, 10nM; Torin 1, 250 nM; Silv: silvestrol, 25 nM, Das: Dasatinib, 100nM; eCF: eCF506, 100nM). Immunoblots (representative of 3 independent experiments) show PRC2 subunit expression and markers of mTORC1 signaling.

Supplementary Figure 6

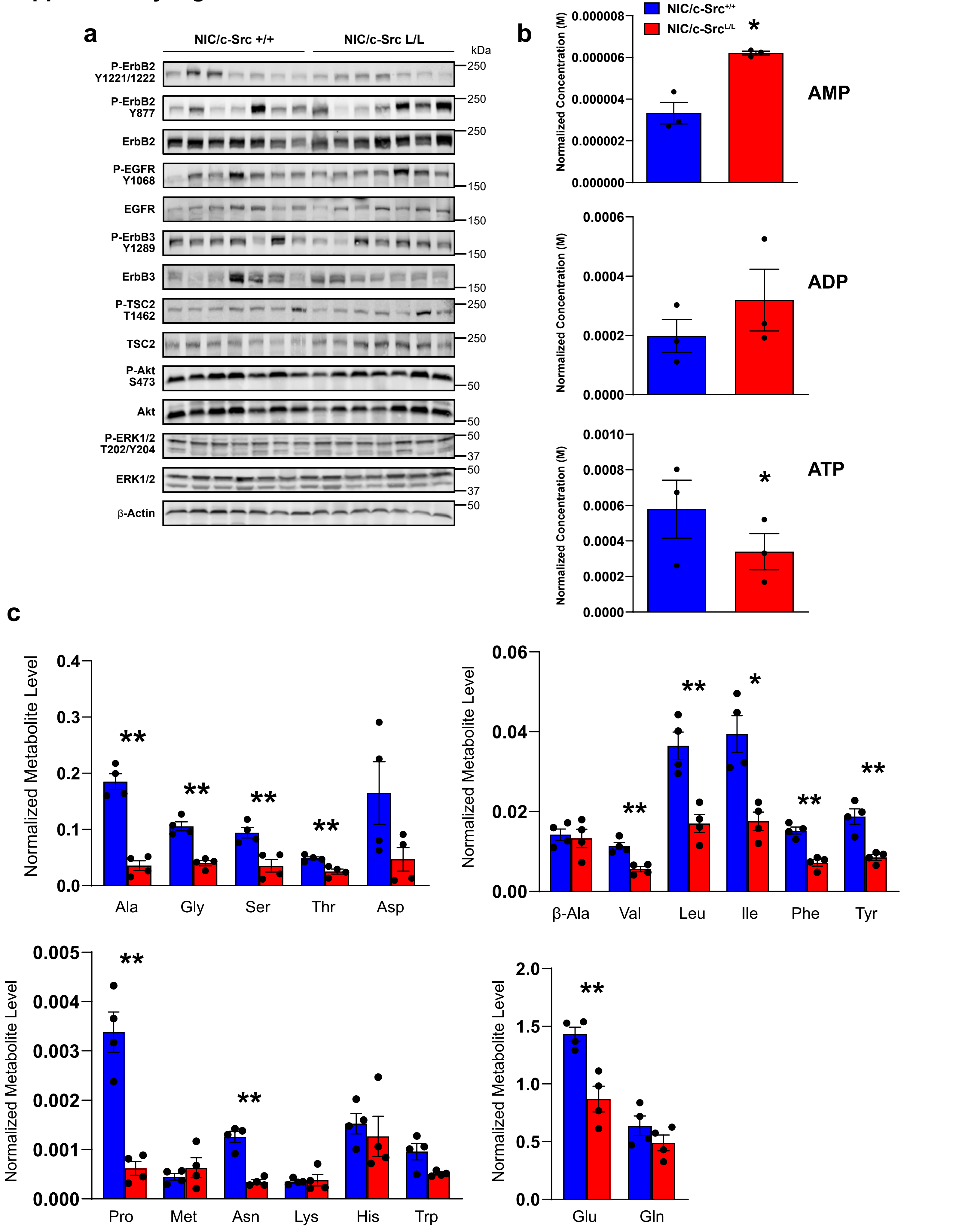

Supplementary Figure 6. mTORC1 inactivation in c-Src-deficient tumours coincides with energy stress and reduced amino acid levels but not changes in canonical RTK signalling. (a) Immunoblots showing the phosphorylation status of ErbB-family RTKs and downstream signaling effectors in NIC/c-Src$^{+/+}$ and NIC/c-Src$^{L/L}$ mammary tumors (seven biological replicates per genotype). **(b)** LC/MS analysis of adenine nucleotide levels in NIC/c-Src$^{+/+}$ and NIC/c-Src$^{L/L}$ cell lines. Data are mean +/- SEM of three cell lines per genotype (*p <0.05, unpaired, two-tailed Student's t-test). **(c)** Steady-state amino acid levels in NIC/c-Src$^{+/+}$ and NIC/c-Src$^{L/L}$ cells were determined using GC/MS (4 cell lines per genotype, mean +/- SEM, *p < 0.05, **p < 0.01; unpaired, two-tailed Student's t-test).

Supplementary Figure 7

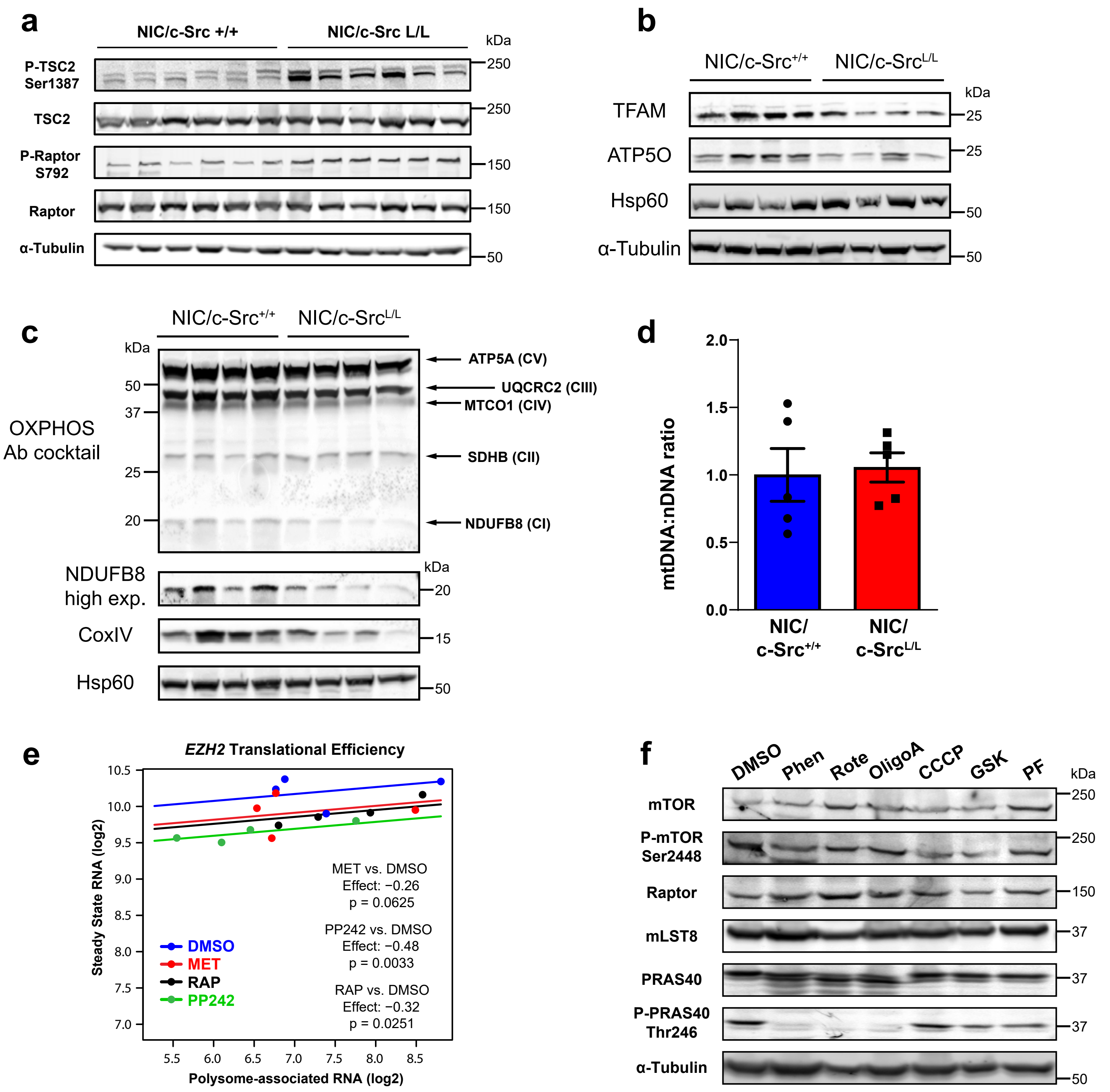

Supplementary Figure 7. AMPK-dependent suppression of mTORC1 in c-Src-deficient cells induces changes in mitochondrial protein expression and loss of *EZH2* translation in human breast cancer cells. **(a)** NIC/c-Src$^{+/+}$ and NIC/c-Src$^{L/L}$ mammary tumors were analyzed by immunoblotting to detect AMPK-dependent phosphorylation of TSC2 (Ser1387) and Raptor (Ser792). α-Tubulin was used as a loading control. **(b-c)** NIC/c-Src$^{+/+}$ and NIC/c-Src$^{L/L}$ cells were lysed and immunoblotted to detect the indicated mitochondrial proteins and α-Tubulin as a loading control. The OXPHOS antibody cocktail contains five primary antibodies against components of each of the electron transport chain complexes. **(d)** Total cellular DNA was isolated from NIC/c-Src$^{+/+}$ and NIC/c-Src$^{L/L}$ cells and the ratio of mitochondrial (mtDNA) to nuclear (nDNA) DNA was determined using QRT-PCR (n=5 per genotype, experiment performed in triplicate). No significant difference was observed (unpaired, two-tailed Student's t-test). **(e)** A published dataset[27] was interrogated to determine polysome-associated and cytoplasmic steady state *EZH2* mRNA levels in MCF7 cells treated with metformin (MET – 10 mM), PP242 (1 μM) or rapamycin (RAP – 100 nM) for 12h. The anota method[75] was used to calculate the effect of each drug on the translational activity of *EZH2* (4 biological replicates). Effects refer to log2 fold-changes in translational activity. Effects and associated p-values were calculated using analysis of partial variance (APV) under a random variance model as implemented in the anota R-package[75]. **(f)** NIC/c-Src$^{+/+}$ cells treated with inhibitors of OXPHOS or AMPK agonists in Figure 5h were analyzed by immunoblotting to detect expression and phosphorylation (for mTOR and PRAS40) of mTORC1 component proteins.

Supplementary Figure 8

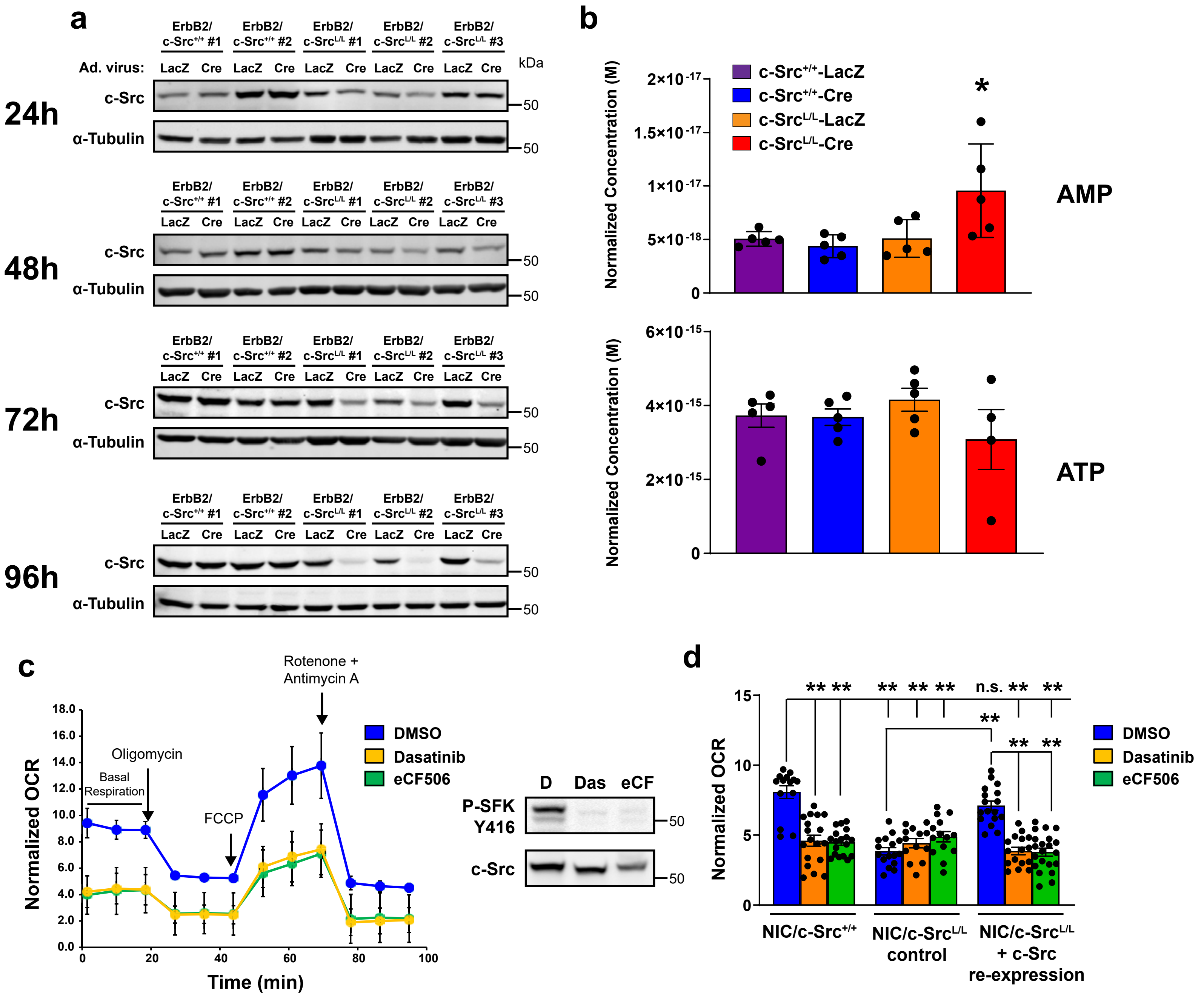

Supplementary Figure 8. Acute ablation or pharmacological inhibition of c-Src reprograms metabolism in ErbB2+ breast cancer cells. (a) ErbB2+ murine breast cancer cells with (ErbB2/c-Src$^{L/L}$ - 3 independent cell lines) or without (ErbB2/c-Src$^{+/+}$ - two independent cell lines) homozygous conditional *Src* alleles were infected in culture with adenoviruses bearing Cre recombinase or LacZ as a negative control. c-Src expression levels were monitored at the indicated times post-infection by immunoblotting. α-Tubulin was used as a loading control. **(b)** LC/MS analysis of AMP and ATP levels in ErbB2/c-Src$^{+/+}$ and ErbB2/c-Src$^{L/L}$ cell lines infected with LacZ- or Cre-expressing adenoviruses. Data are mean +/- SEM for two biological replicates analyzed in quadruplicate (*p <0.05, unpaired, two-tailed Student's t-test). **(c)** Basal, maximal (FCCP), ATP-synthesis-coupled (Oligomycin A), and non-mitochondrial (rotenone/antimycin A) oxygen consumption rates (OCRs) of NIC/c-Src$^{+/+}$ cell lines treated with the Src family kinase inhibitors dasatinib or eCF506 or vehicle control (DMSO) for 24h. Representative of 3 cell lines per genotype, analyzed in quadruplicate. Right panel shows immunoblots indicating expression of activated Src family kinases and total c-Src (D - DMSO, Das - Dasatinib, 100nM, eCF - eCF506, 100nM). **(d)** Quantification of basal oxygen consumption rates of NIC/c-Src$^{+/+}$ and NIC/c-Src$^{L/L}$ cells treated with DMSO, Dasatinib or eCF506 (both 100nM) for 24h. NIC/c-Src$^{L/L}$ cells were additionally transduced with a retrovirus expressing c-Src or a control retrovirus as in Supplementary Fig. 4i. Mean +/- SEM, brackets represent comparisons of conditions to the indicated DMSO control (blue bar). OCR in NIC/c-Src$^{L/L}$ cells reconstituted with c-Src and treated with DMSO was not significantly different from that of NIC/c-Src$^{+/+}$ cells treated with DMSO. All other comparisons not indicated were not signficant (*p < 0.05, **p < 0.01, n.s. not significant; One-way ANOVA with Dunnett's post-hoc test).

Supplementary Figure 9

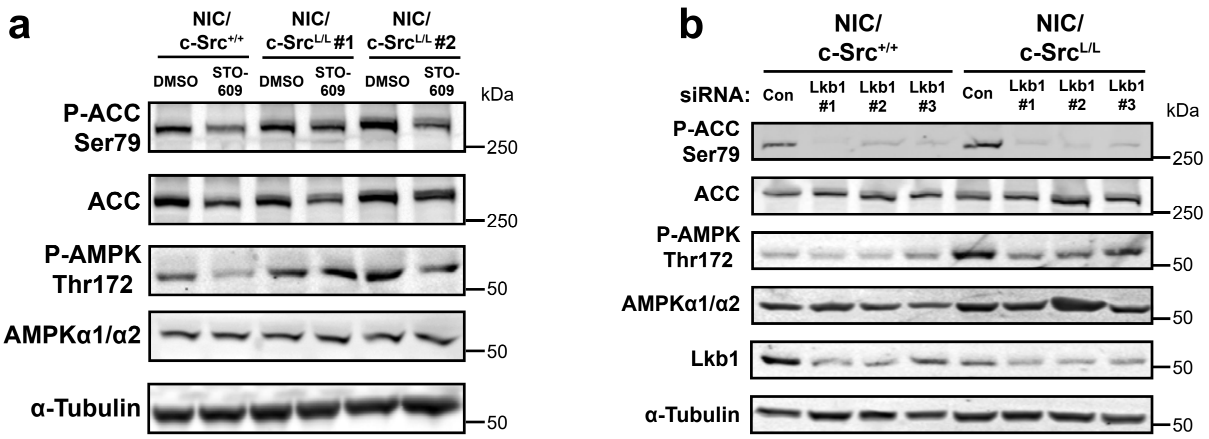

Supplementary Figure 9. Lkb1 and CAMKKβ contribute to basal AMPK activation in ErbB2+ breast cancer cells and to the increase in AMPK activity in c-Src-deficient ErbB2+ breast cancer cells. (a) NIC/c-Src$^{+/+}$ and NIC/c-Src$^{L/L}$ cells were treated with the CAMKKβ inhibitor STO-609 (10μM) for 24h and immunoblotted to detect the indicated proteins. **(c)** NIC/c-Src$^{+/+}$ and NIC/c-Src$^{L/L}$ cells were transfected with siRNAs targeting Lkb1 or a control siRNA (Con) as indicated and lysed 72h after transfection. Immunoblotting was used to detect the indicated proteins.

Supplementary Figure 10

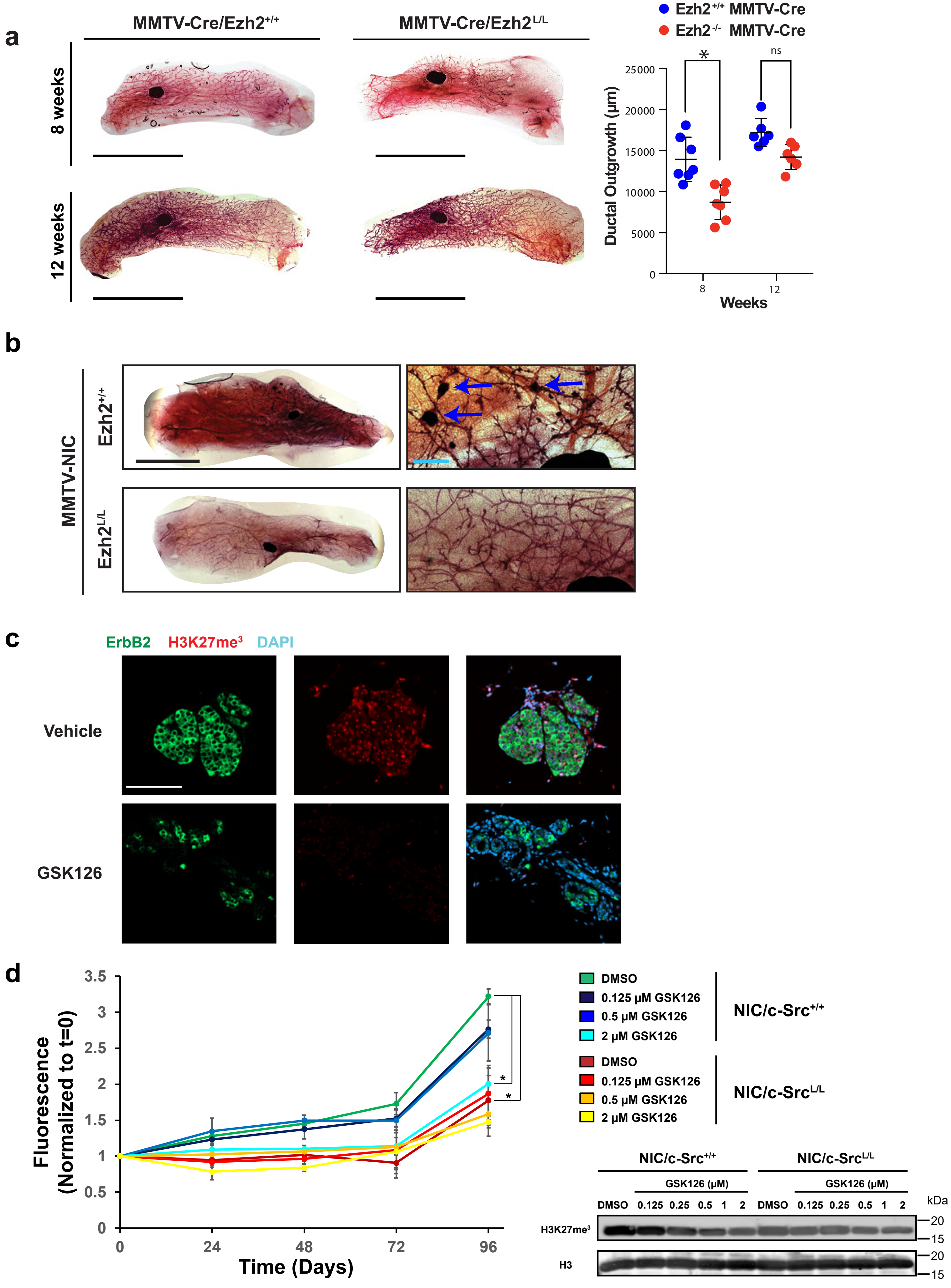

Supplementary Figure 10. Ezh2 ablation causes a transient delay in mammary ductal outgrowth and blocks the formation of neoplastic lesions in the mammary epithelium. (a) Left - L4 mammary glands from 8- and 12-week old MMTV-Cre/Ezh2$^{+/+}$ and MMTV-Cre/Ezh2$^{L/L}$ mice (n=6 per genotype) were whole-mounted and stained with hematoxylin. Right – scatter plot indicates ductal outgrowth as determined by measuring the distance from the lymph node to the terminal end buds. *$p < 0.05$, unpaired Student's t-test; n.s., not significant. Scale bar represents 5 mm. **(b)** Whole-mounted mammary glands from 16 week-old NIC/Ezh2$^{+/+}$ and NIC/Ezh2$^{L/L}$ mice. Arrows indicate neoplastic mammary epithelial lesions in NIC/Ezh2$^{+/+}$ mice. Images are representative of whole-mounts from four independent mice of each genotype. Scale bars represent 5000 µm (left images) or 1000 µm (right images). **(c)** Immunofluorescence (IF) was used to detect H3K27me^3 in ErbB2-positive mammary epithelial cells of MMTV-NIC mice treated with GSK126 or vehicle control. Images representative of six mice per genotype, scale bar indicates 100 µm. **(d)** NIC/c-Src$^{+/+}$ and NIC/c-Src$^{L/L}$ cells (n=4 cell lines per genotype) were treated with the EZH2 inhibitor GSK126 at the indicated concentrations and proliferation was assessed. (*$p<0.05$, one-way ANOVA with Tukey's post-test). Lower right panel is a representative immunoblot showing inhibition of H3K27 tri-methylation.

Supplementary Figure 11

Uncropped Immunoblots

Red boxes indicate approximate areas cropped in the figures

Figure 2 (Note that blots in Fig. 2B are uncropped)

Fig. 2A

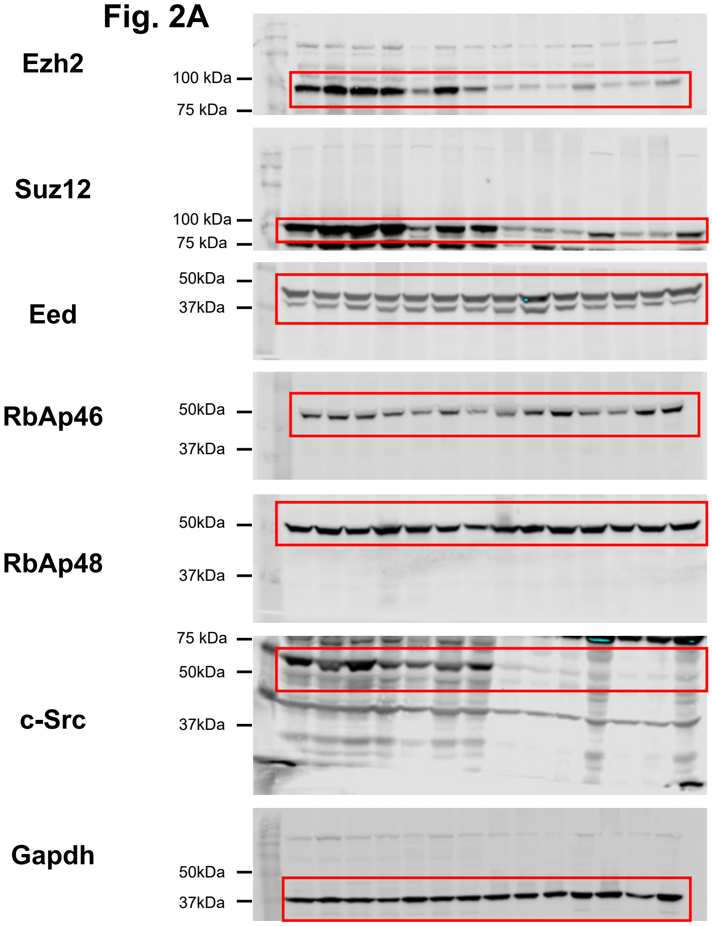

Fig. 2C

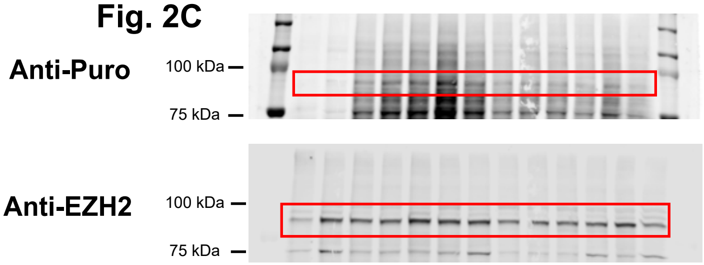

Supplementary Figure 11

Fig. 3B

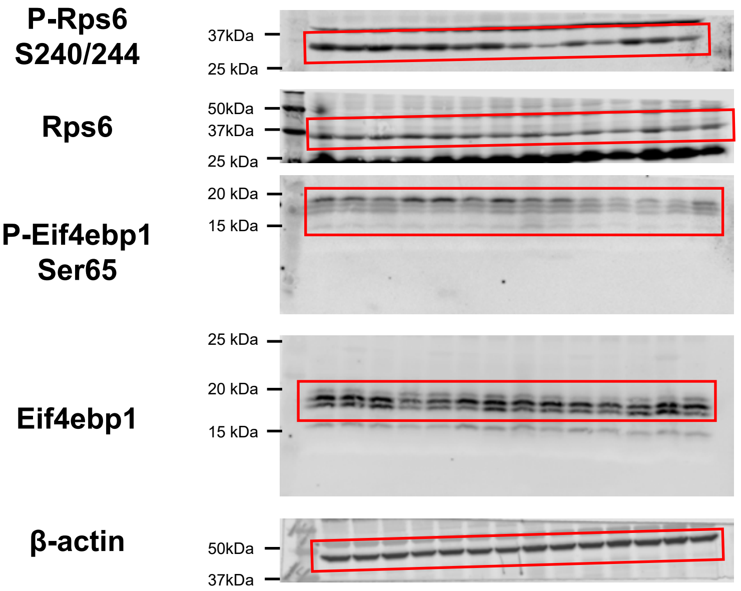

Fig. 3C

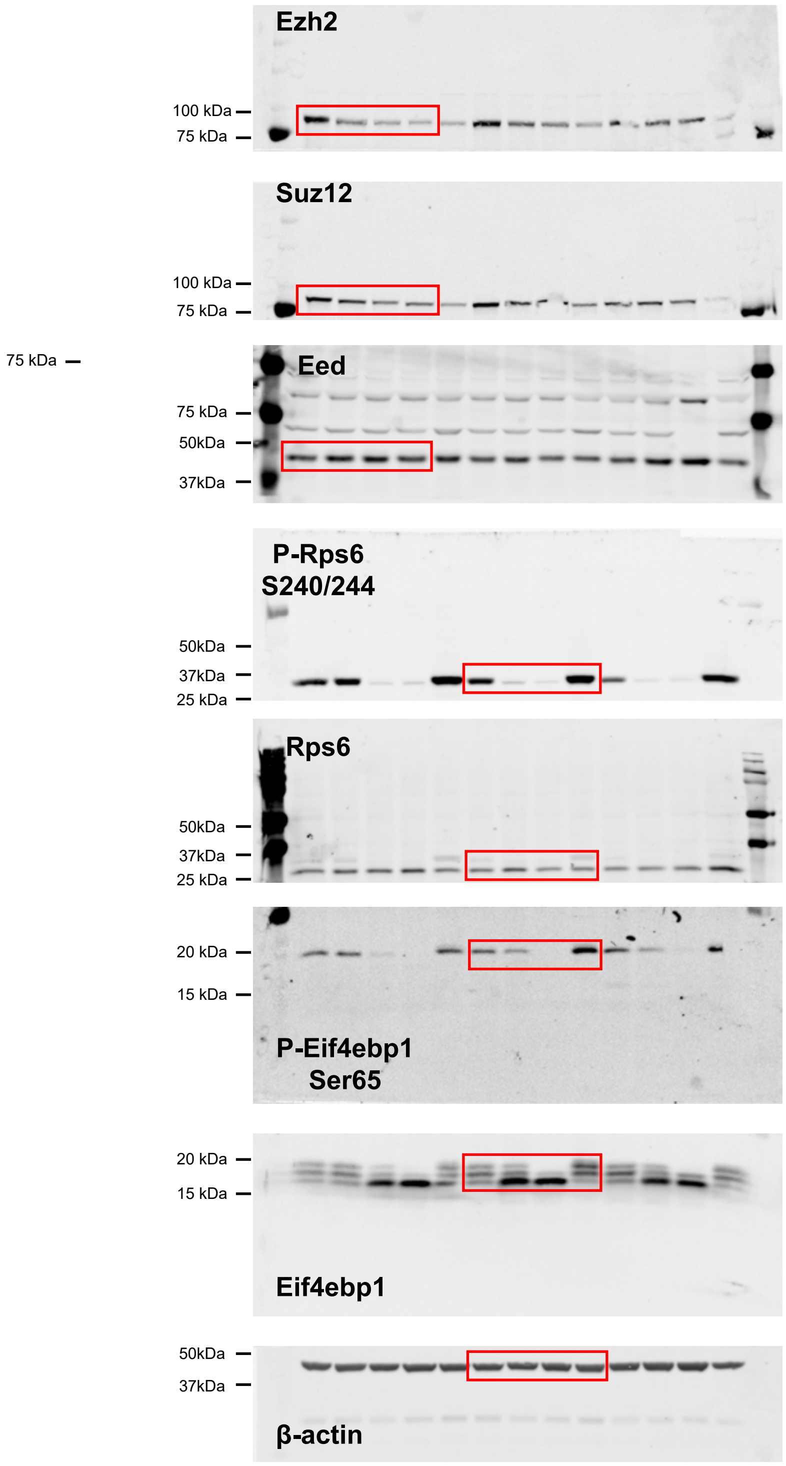

Supplementary Figure 11

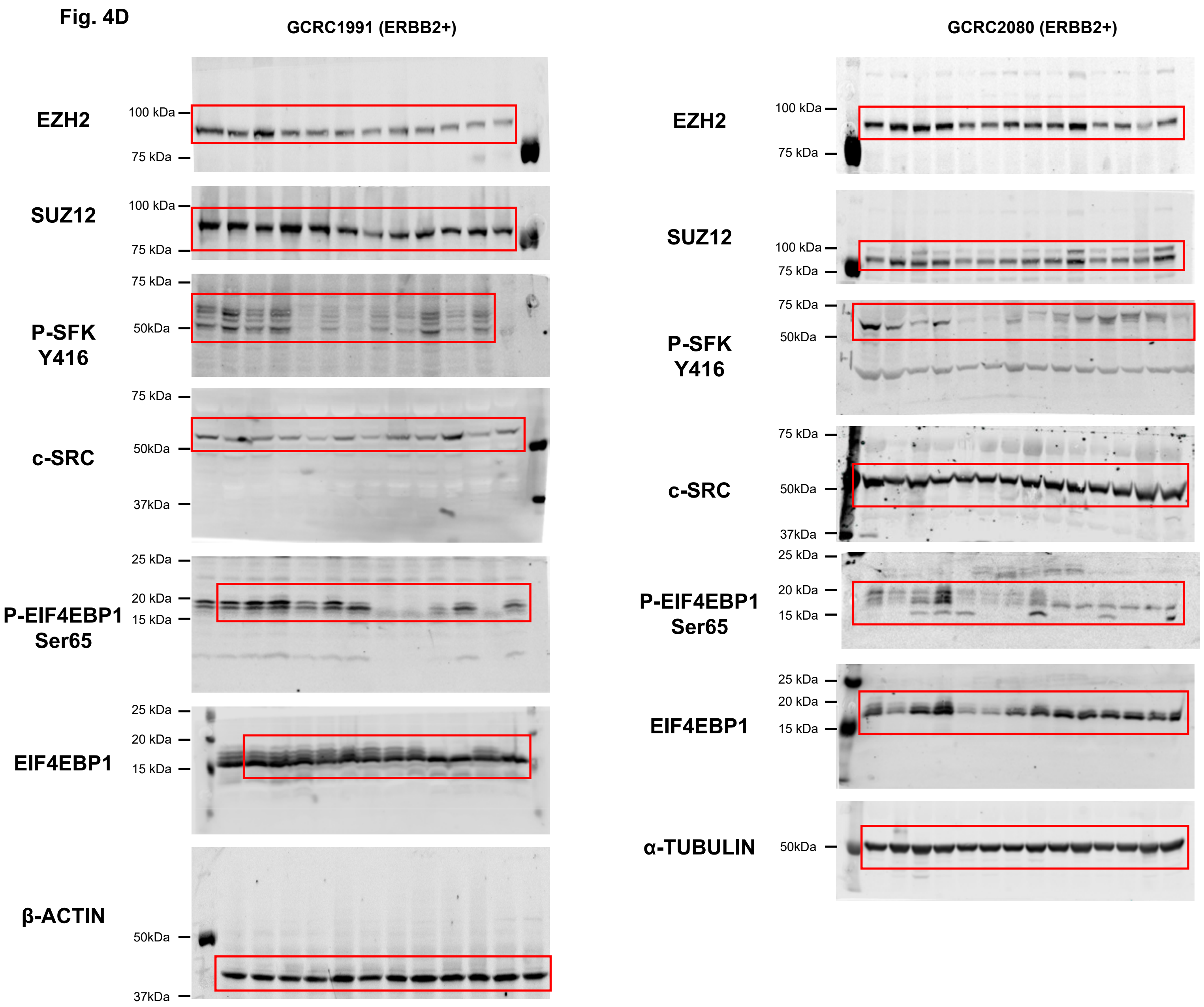

Supplementary Figure 11

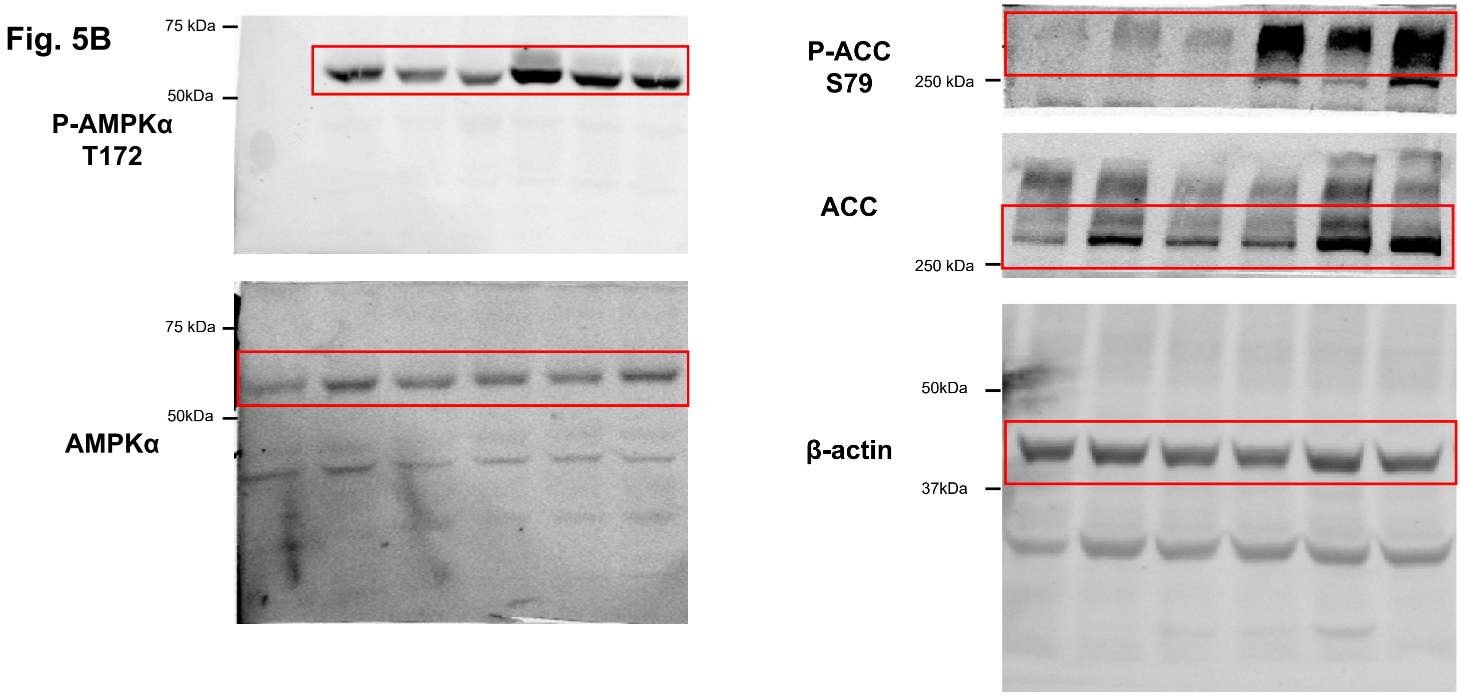

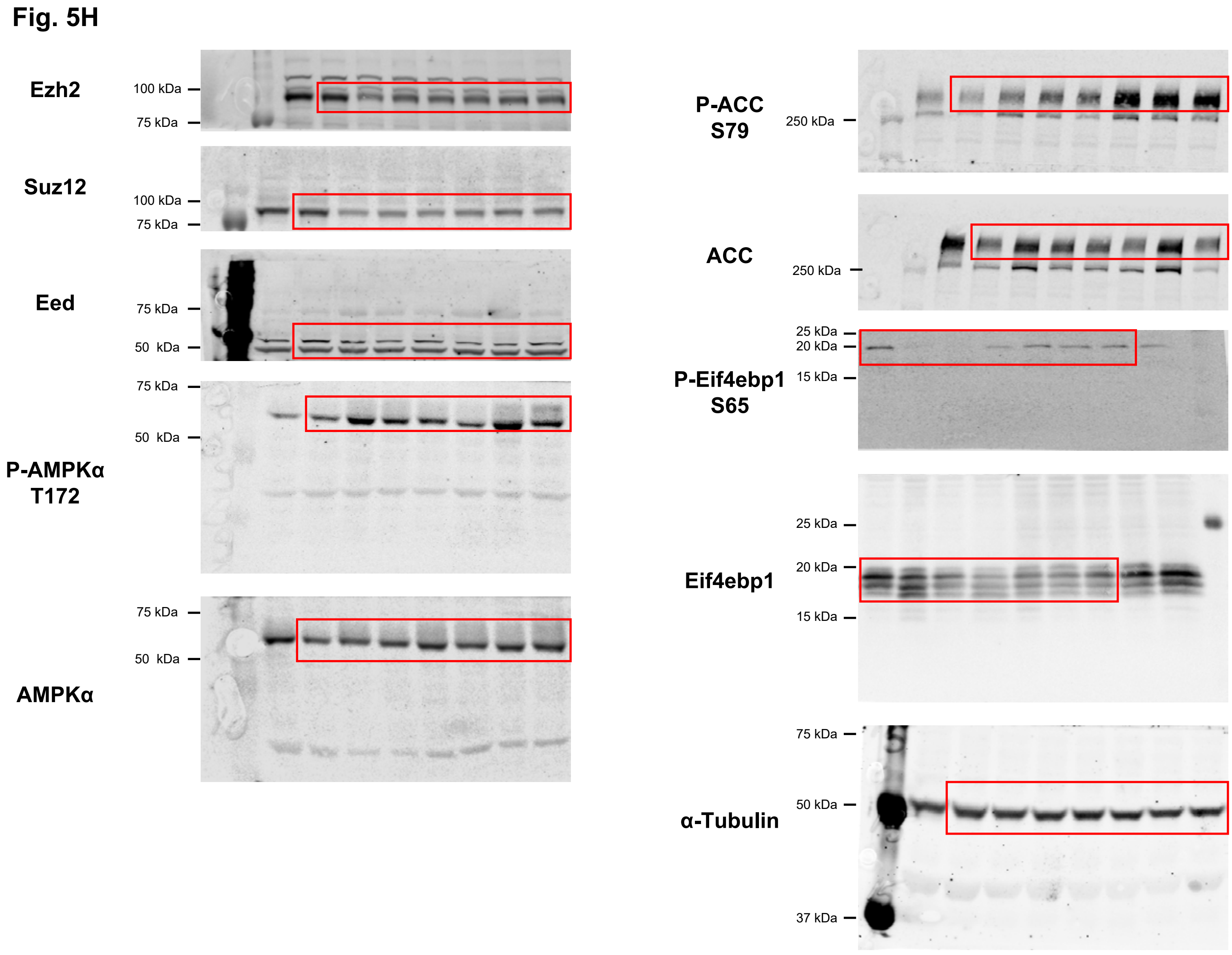

Supplementary Figure 11

Fig. 6A

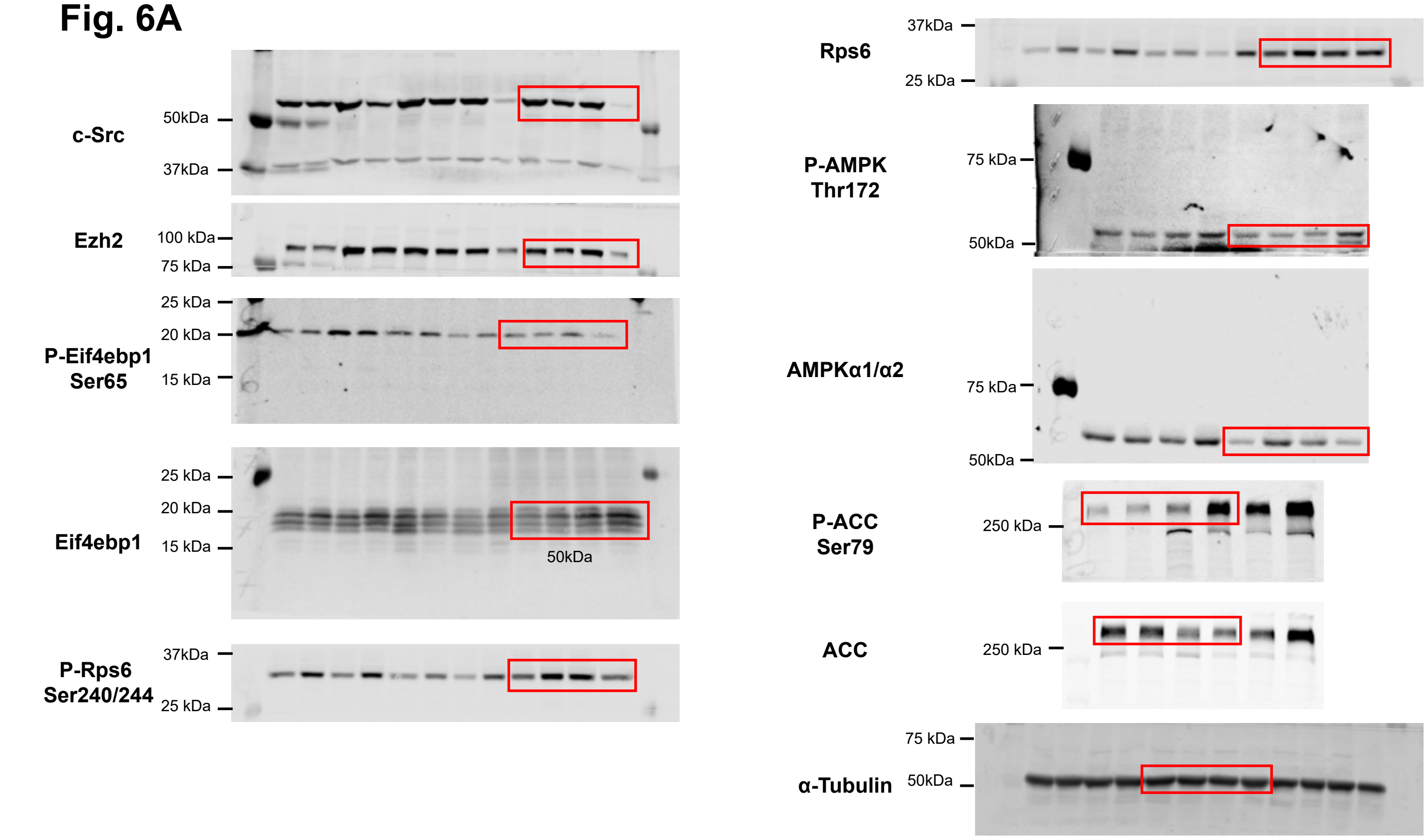

Fig. 7A

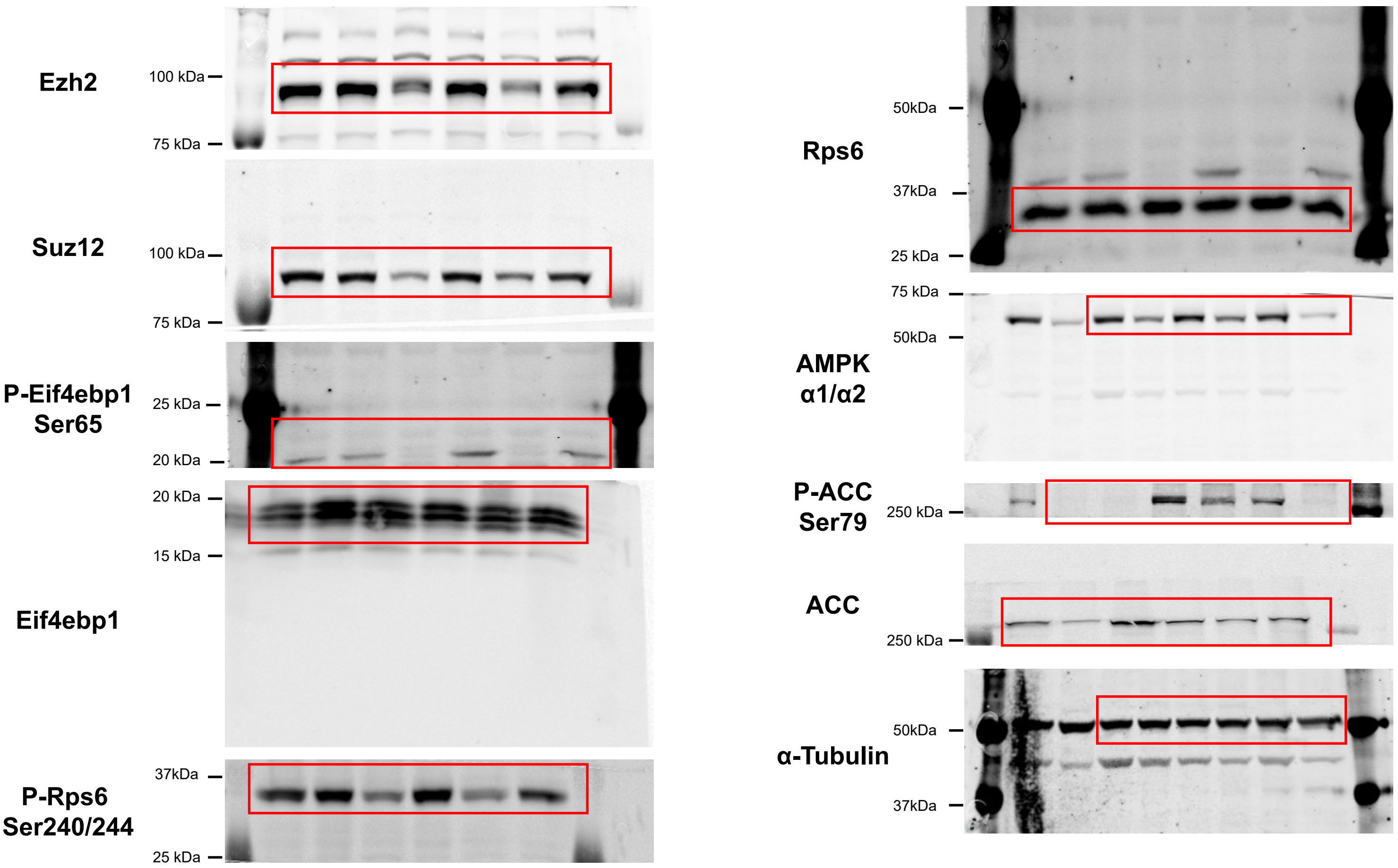

Supplementary Figure 11

Fig. 9A

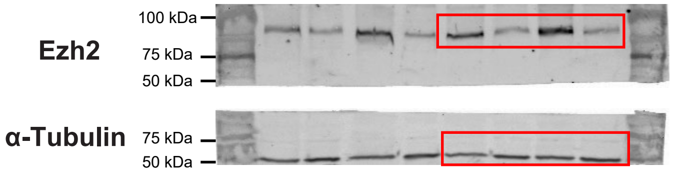

Fig. 9C

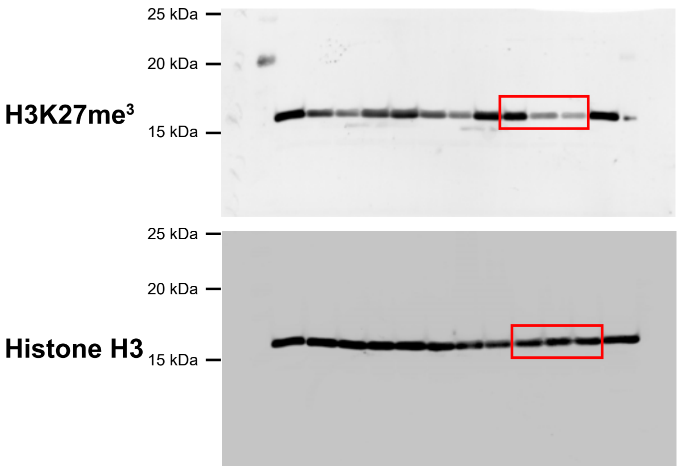

Supplement: Supplementary file 1 — Supplementary Information [file 41467_2019_10681_MOESM1_ESM.pdf]
