# Supplementary material for: An ErbB2/c-Src axis links bioenergetics with PRC2 translation to drive epigenetic reprogramming and mammary tumorigenesis
Source: Nat Commun. 2019 Jul 1;10:2901. doi: 10.1038/s41467-019-10681-4 (PMC6603039; doi:10.1038/s41467-019-10681-4)
Supplement: Supplementary file 2 — Description of Additional Supplementary Files [file 41467_2019_10681_MOESM2_ESM.docx]

Description of Additional Supplementary Files

**Supplementary Data 1:** Oligonucleotides and primers used in this study. This table lists all primers used for QRT-PCR or genotyping, plasmids used for lentivirus/retrovirus production and siRNA oligonucleotides. Catalogue numbers and/or sequences are provided.
